# Germinated Riceberry Rice Enhanced Protocatechuic Acid and Vanillic Acid to Suppress Melanogenesis through Cellular Oxidant-Related Tyrosinase Activity in B16 Cells

**DOI:** 10.3390/antiox9030247

**Published:** 2020-03-19

**Authors:** Teerapat Rodboon, Seiji Okada, Prasit Suwannalert

**Affiliations:** 1 Department of Pathobiology, Faculty of Science, Mahidol University, Bangkok 10400, Thailand; rteerapat@gmail.com; 2 Division of Hematopoiesis, Joint Research Center for Human Retrovirus Infection & Graduate School of Medical Sciences, Kumamoto University, Kumamoto 860-0811, Japan; okadas@kumamoto-u.ac.jp

**Keywords:** melanogenesis, riceberry rice, antioxidants, tyrosinase, oxidative stress

## Abstract

The anti-melanogenic bioactivities of phytophenolic compounds have been well recognized. Riceberry rice contains a rich source of phenolic compounds that act as melanin inhibitors through their antioxidant and anti-tyrosinase properties. Germination has been shown to be an effective process to improve targeted phenolic compounds. In this study, germinated riceberry rice extract was tested for antioxidant activity. Total phenolic content was determined while the tyrosinase inhibitory effect was screened by the in vitro mushroom tyrosinase assay. Cytotoxicity of germinated riceberry rice extract was investigated in B16 cells before evaluating its activities on cellular tyrosinase, melanogenesis, melanin excretion, morphological appearance, and cellular oxidants. Germinated riceberry rice extract showed increased potency of antioxidants and was also twice as effective for mushroom tyrosinase inhibition when compared with ungerminated riceberry rice extract. In B16 cells, the extract inhibited cellular tyrosinase, melanogenesis, and cellular oxidants in a dose-dependent manner when compared with untreated cells. Germinated riceberry rice extract also displayed an effect on B16 cells morphology by reducing the number of melanin- containing cells and their dendriticity. Additionally, the germination of riceberry rice dominantly enhanced two phenolic acids, protocatechuic acid and vanillic acid, which have the potential for antioxidant-associated hyperpigmentation control. Thus, the restricted germination of riceberry rice tended to promote protocatechuic acid and vanillic acid, which dominantly displayed antioxidants and tyrosinase-related melanogenic inhibition.

## 1. Introduction

Hyperpigmentation disorder results from the overproduction of melanin pigments and has been identified as a dermatological problem that can be psychologically devastating to patients [1]. A wide variety of chemical agents have been used extensively for hyperpigmentation control. Several reports have examined their adverse effects and toxicity [2,3]. In this study, novel effective natural products for hyperpigmentation treatment were elucidated. Several phenolic acids have been identified as having anti-melanogenic properties via the inhibition of tyrosinase, a key enzyme for melanogenesis [4,5,6,7]. In addition, the antioxidant activity of natural compounds was also considered for the protection and treatment of hyperpigmentation [8,9].

Riceberry rice, a new breeding line of Thai black purple rice, has shown high levels of antioxidants and targeted phenolic compounds including ferulic acid, gallic acid, 4-hydroxybenzoic acid, vanillic acid, chlorogenic acid, syringic acid, p-coumaric acid, and ferulic acid [10,11] that act as tyrosinase inhibitors. Previously, a suitable germination process promoted both the quantitative and qualitative aspects of these valuable phenolic compounds [12,13,14,15]. In this study, riceberry rice was enhanced for anti-melanogenesis by the germination process. Germinated riceberry rice extract could be further developed as a novel agent for hyperpigmentation control.

## 2. Materials and Methods

### 2.1. Rice Germination

Riceberry rice was obtained from Phayao province, Thailand. A hundred grams of rice were soaked in reverse osmosis water, pH 7.2–7.4 for 1 h. Soaked riceberry rice was germinated in a polyethylene tray (22 × 22 × 5 cm) at room temperature in the dark for 6 h. Subsequently, germinated riceberry rice was lyophilized and then ultracentrifugally powdered. The riceberry rice powder was then sieved through 345 μm and stored at −20 °C until extraction.

### 2.2. Rice Sample Extraction

Riceberry rice powder was extracted by using 80% ethanol in distilled water at a ratio of 1:4. The mixture was continuously shaken at 10 g, 25 °C for 6 h under dark conditions. The supernatant was harvested by centrifugation at 800× *g* for 15 min and sterilized by filtration through Whatman Filter Paper No.1. The sample was dried using a rotary evaporator (Marshall Scientific, Hampton, VA, USA) at 40 °C, 200 mm/Hg, and dehydrated by a lyophilizer (Thermo Fisher Scientific, Waltham, MA, USA). Dried stock samples were stored at −20 °C until used in the experiments.

### 2.3. In Vitro Antioxidant Activities

#### 2.3.1. 2,2′-Azino-bis(3-Ethylbenzothiazoline-6-Sulfonic Acid) (ABTS) Radical Scavenging Assay

The ABTS radical scavenging activity of the sample was investigated by using a modified method from a previous study [16]. Briefly, the ABTS• stock solution (7 mM) was prepared by reacting ABTS (Sigma-Aldrich, Singapore) with 2.45 mM potassium persulfate (Sigma-Aldrich, Singapore) solution at room temperature under dark conditions for 12 h. A working ABTS• solution was then prepared by diluting the stock solution with distilled water. The working reagent was adjusted to obtain an absorbance of 0.80 ± 0.05 at 734 nm. Then, 200 μL of sample or Trolox (Sigma-Aldrich, Singapore) were reacted with 1.8 mL of working ABTS• solution for 30 min before measuring at 734 nm. The ABTS radical scavenging activity was calculated and compared with the Trolox standard curve. The result was expressed as mg Trolox equivalent/g sample.

#### 2.3.2. 2,2 Diphenyl-1-Picrylhydrazyl (DPPH) Radical Scavenging Assay

The DPPH radical scavenging activity of the sample was examined as previously mentioned [17]. Briefly, a working DPPH• solution (6 μM) was freshly prepared by adjusting the absorbance of 2 mM DPPH• (Sigma-Aldrich, Singapore) stock solution to 0.80 ± 0.05 at 517 nm by using methanol. Then, 200 μL of sample at various concentrations or a serial dilution of L-ascorbic acid (VitC) (Sigma-Aldrich, Singapore) were reacted with 1.8 mL of working DPPH• solution for 30 min before measuring at 517 nm. The DPPH radical scavenging activity was calculated and compared with the activity with the Vit C standard curve. The result was expressed as mg Vit C equivalent/g sample.

#### 2.3.3. Ferric Reducing Antioxidant Power (FRAP) Assay

The FRAP assay is a method for determining the antioxidant activity of a sample to reduce Fe^3+^ to Fe^2+^. This assay was carried out according to a previous study [18]. Briefly, the FRAP reagent was freshly prepared by mixing 300 mM acetate buffer with 10 mM TPTZ (Sigma-Aldrich, Singapore) solution and 20 mM ferric chloride (Sigma-Aldrich, Singapore) solution. Then, 180 μL of FRAP reagent were reacted with 20 μL of sample or FeSO_4_·7H_2_O (Sigma-Aldrich, Singapore) for 30 min before measuring at 593 nm. Ferric reducing activity was calculated and the activity compared with the FeSO_4_·7H_2_O standard curve. The result was expressed as M FeSO_4_·7H_2_O equivalent/g sample.

### 2.4. Total Phenolic Content

The total phenolic content of the sample was determined by the Folin–Denis assay, as described previously [8]. Briefly, 20 μL of sample or gallic acid (Sigma-Aldrich, Singapore) were mixed with 100 μL of Folin–Denis reagent (Sigma-Aldrich, Singapore) and 1880 μL of 7.5% aqueous sodium bicarbonate solution for 30 min at room temperature. The reaction was measured at 765 nm. The total phenolic content was calculated and the activity compared with the gallic acid standard curve. The result was expressed as mg gallic acid equivalent/g sample.

### 2.5. In Vitro Mushroom Tyrosinase Activity

The inhibitory effect of the sample for tyrosinase inhibition was investigated by an in vitro mushroom tyrosinase activity assay modified from a previous study [19]. Briefly, 50 μL of sample were pre-incubated with 100 μL of phosphate buffered saline (PBS), pH 7.1, and 50 μL of mushroom tyrosinase (Sigma-Aldrich, Singapore) at room temperature for 10 min. The mixture was then incubated with 20 μL of 1 mM 3,4-dihydroxyphenylalanine (L-DOPA) (Sigma-Aldrich, Singapore) at 37 °C for 30 min. Dopachrome was measured at 492 nm. The result was expressed as the necessary concentration to achieve 50% inhibition (IC_50_).

### 2.6. Cell Culture

B16 cells (ATCC Number CRL-6475^TM^, VA, USA) were maintained in low glucose DMEM (Sigma-Aldrich, Singapore) supplemented with 10% fetal bovine serum (Gibco Life Technologies, Massachusetts, MA, USA), 10 U/mL of penicillin (Gibco Life Technologies, Massachusetts, MA, USA), and 10 mg/mL of streptomycin (Gibco Life Technologies, Massachusetts, MA, USA) in a 37 °C, 5% CO_2_, and 95% humidified atmosphere.

### 2.7. Cell Viability 

#### 2.7.1. MTT 3-(4,5-Dimethyl-2-yl)-2,5-Diphenyltetrazolium Bromide Assay

The cytotoxicity of the sample on B16 cells was measured by using the MTT assay modified from a previous study [20]. Briefly, B16 cells were plated into 96 well plates at a density of 3 × 10^4^ cells/well and then incubated for 24 h. B16 cells were treated with the sample at various concentrations and incubated for 24 h. Treated cells were then reacted with 1.2 mM MTT (Sigma-Aldrich, Singapore) solution for 3 h. The formazan crystal was then solubilized with absolute DMSO (Merck, Kenilworth, NJ, USA) and measured at 530 nm. The result was expressed as the percentage of cell viability by comparing the mean ± SD with untreated cells (100% of cell viability).

#### 2.7.2. Trypan Blue Viability Assay

Cell viability was also determined by the trypan assay, based on a previous study [21]. Briefly, 3 × 10^4^ of B16 cells were plated into 96 well plates and incubated for 24 h. The cells were treated with the sample at various concentrations and further incubated for 24 h. The cells were detached by trypsinization and stained by 0.1% trypan blue solution (Merck, Kenilworth, NJ, USA). The viable cells and dead cells were counted separately, and the percentage of viable cells was calculated by comparing the mean ± SD with untreated cells (100% of cell viability).

### 2.8. Melanin Content and Melanin Excretion

Melanin content was determined as described previously [22]. Briefly, 1.0 × 10^5^ of B16 cells were plated into 6 well plates and incubated for 24 h. The cells were treated with non-toxic concentrations of the sample and incubated for 72 h. Melanin excretion was optically determined in a culture medium at 405 nm. Intracellular melanin content was detected in pellets by disruption with 1 N NaOH solution at 80 °C for 4 h and measured at 405 nm. Ten micromolar hydroquinone (HQ) (Sigma-Aldrich, Singapore) was used as a positive control. 

### 2.9. Differentiation and Morphological Appearances of B16 Cells

The effects of the samples on differentiated B16 cells and their morphological appearances were observed using a modified of Fontana–Masson staining method [23]. Briefly, 1.0 × 10^5^ of B16 cells were plated into 6 well plates and incubated for 24 h. Cells were treated with the sample at the indicated concentrations and further incubated for 72 h. Fontana–Masson staining was conducted by using a Fontana–Masson Stain Kit (Bio-Optica, Milan, Italy) according to the manufacturer’s instructions. The number of cells containing melanin was counted in a total of 1000 of B16 cells under a light microscope at 100× magnification. The morphological appearances of differentiated B16 cells were scored in a total of 100 differentiated B16 cells as 1+, 2+, 3+, and 4+, as presented Table 1.

### 2.10. Cellular Tyrosinase Activity

The effect of samples on cellular tyrosinase activity was determined using a method mentioned previously [24]. Briefly, B16 cells were plated into 6 well plates at a density of 1.0 × 10^5^ cells/mL and incubated for 24 h. The cells were treated with the sample at non-toxic concentrations and incubated for 72 h. Treated cells were solubilized with PBS, pH 7.1 containing 1% Triton X-100 (Sigma-Aldrich, Singapore). The cell lysate was clarified by centrifugation at 15,000× *g* for 15 min to obtain the supernatant. Then, 50 μL of supernatant were reacted with 50 μL of 2 mM L-DOPA at 37 °C for 90 min before measuring the dopachrome product at a wavelength of 492 nm. Ten micromolar hydroquinone (HQ) was used as a positive control. 

### 2.11. Cellular Oxidants

Cellular oxidants of B16 cells were determined by using the dichloro-dihydro-fluorescein diacetate (DCFH-DA) method based on a previous study [6]. Briefly, B16 cells were plated into 96 well plates at a density of 3.0 × 10^4^ cells/well and incubated for 24 h. Cells were treated with non-toxic concentrations of the sample and incubated for 24 h. The cells were washed with PBS, pH 7.4, and incubated with 10 μM DCFH-DA (Merck, Kenilworth, NJ, USA) solution for 3 h. The cellular oxidants were determined by fluorescence intensity at 485/535 nm. Twenty millimolar N-acetylcysteine (NAC) (Merck, Kenilworth, NJ, USA) was used as a positive control. 

### 2.12. High Performance Liquid Chromatography (HPLC)

Phytophenolics in the samples were identified using an HPLC technique with slight modification [25]. Briefly, samples were eluted by absolute acetonitrile solution (A) and 0.1% trifluoroacetic acid solution in distilled water (B) under the gradient elution (Solvent A:Solvent B) at 5–9%:95–91%, 9%:91%, 9–11%:91–89%, and 11–50%:89–50% with respective time periods of 0–5, 5–15, 15–22, and 22–25 min. The flow rate was controlled at 0.8 mL/min under an ACE^®^ C18 column (Advanced Chromatography Technologies, Aberdeen, Scotland) (250 mm × 4.6 mm; 5 μm) at a temperature of 40 °C. The fingerprints of phenolic acids were detected with a UV detector at 280 nm. The commercial standard of protocatechuic acid (Sigma-Aldrich, Singapore) and vanillic acid (Sigma-Aldrich, Singapore) were designed for fingerprint identification.

### 2.13. Statistical Analysis

Data were expressed as the mean ± SD. The paired Student *t*-test and one-way ANOVA followed by Turkey’s test were used for the determination of the difference between the groups and the control. Significance was considered at *p* ≤ 0.05. 

## 3. Results

### 3.1. Antioxidant Activity, Phenolic Content, and Mushroom Tyrosinase Inhibition of Germinated Riceberry Rice Extract

Antioxidants are accepted for use in improving hyperpigmentation. In this study, the germination of riceberry rice promoted the antioxidant activity in all in vitro tests, while total phenolic content was not significantly different (Table 2). In addition, germinated riceberry rice extract efficiently inhibited tyrosinase, the key melanogenic enzyme, more than twice as effectively when compared with the an ungerminated sample, suggesting the potential of germinated riceberry rice for hyperpigmentation control.

### 3.2. Inhibitory Effect on Tyrosinase-Related Melanogenesis of Germinated Riceberry Rice Extract Treated B16 Cells

**Figure 1:**
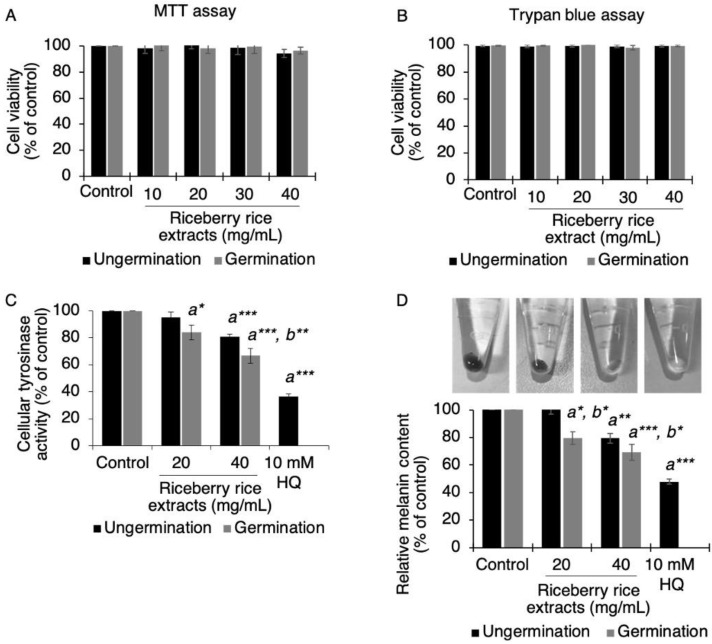
Effects of germinated riceberry rice extract on cellular tyrosinase and total melanin content in B16 cells. B16 cells were treated with ungerminated and germinated riceberry rice extracts at concentrations of 10, 20, 30, and 40 mg/mL and then tested for cytotoxicity by the MTT assay (**A**) and the trypan blue assay (**B**). Non-cytotoxic doses at 20 and 40 mg/mL were used on B16 cells for cellular tyrosinase activity (**C**) and melanin content (**D**). All results were expressed as the mean ± SD by comparing with 100% of untreated cells. Ten millimolar of hydroquinone (HQ) was used as a positive control. *, **, *** Statistical significance at *p* < 0.05, *p* < 0.01, and *p* < 0.001 and *^a, b^* versus untreated cells and the ungerminated riceberry rice treatment group, respectively. Results from triplicate assays of two independent experiments.

The effects of germinated riceberry rice extract on tyrosinase-related melanin inhibition were then investigated at the cellular level using B16 cells. Firstly, the cytotoxic effect of the rice extracts was determined and showed no cytotoxicity at the concentrations of 10–40 mg/mL (Figure 1A,B). At non-toxic concentrations of 20 and 40 mg/mL, germinated riceberry rice extract significantly inhibited cellular tyrosinase activity compared to ungerminated to 84 ± 5.499% (*p* = 0.026) and 67 ± 3.147% (*p* < 0.001), respectively, when compared with untreated cells (100%) (Figure 1C). The result was interrelated with the melanin inhibition. Germinated riceberry rice extract also provided greater activity for cellular melanin inhibition than ungerminated riceberry rice by significantly reducing the melanin content to 80 ± 5.718% (*p* = 0.05) and 69 ± 3.512% (*p* < 0.001), respectively, when compared with untreated cells (100%) (Figure 1D). 

### 3.3. Melanin Pigmentation of B16 Cells Treated with Germinated Riceberry Rice Extract 

**Figure 2:**
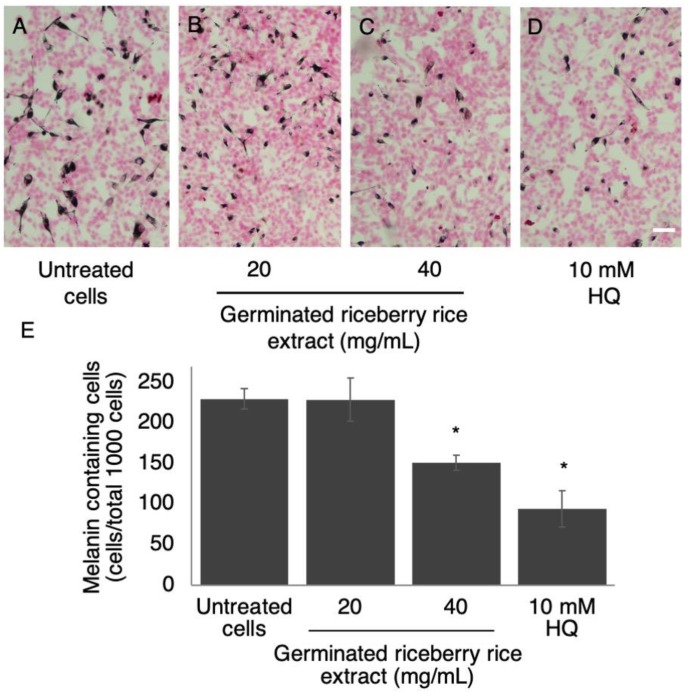
Effect of germinated riceberry rice extract on melanin pigmentation in B16 cells. Fontana–Masson’s staining of B16 cells (**A**), B16 cells treated with germinated riceberry rice extract at 20 (**B**) and 40 mg/mL (**C**), and 10 mM hydroquinone (HQ) (**D**). Melanin containing cells were counted by observing a total of 1000 cells under a light microscope at 100× magnification. Results were expressed as the mean ± SD from triplicate samples from two independent experiments (**E**). * Statistical significances at *p* < 0.001 when compared with untreated cells. Scale bar = 100 μm.

Cellular melanin content is relatively affected by the number of pigmented cells. In this study, the number of melanin containing B16 cells was investigated by using Fontana–Masson staining (Figure 2A–D). Treatment with germinated riceberry rice extract at the concentration of 40 mg/mL significantly reduced the number of melanin containing cells to 133 ± 7.789 cells/total 1000 cells when compared with the untreated cells (230 ± 12.710 cells/total 1000 cells). Additionally, the morphological appearance of B16 cells treated with germinated riceberry rice was also affected at the concentration of 20 mg/mL of treatment. Thus, the morphology of differenced B16 cells was then specifically investigated. 

### 3.4. Morphological Appearance of B16 Cells Treated with Germinated Riceberry Rice Extract

**Figure 3:**
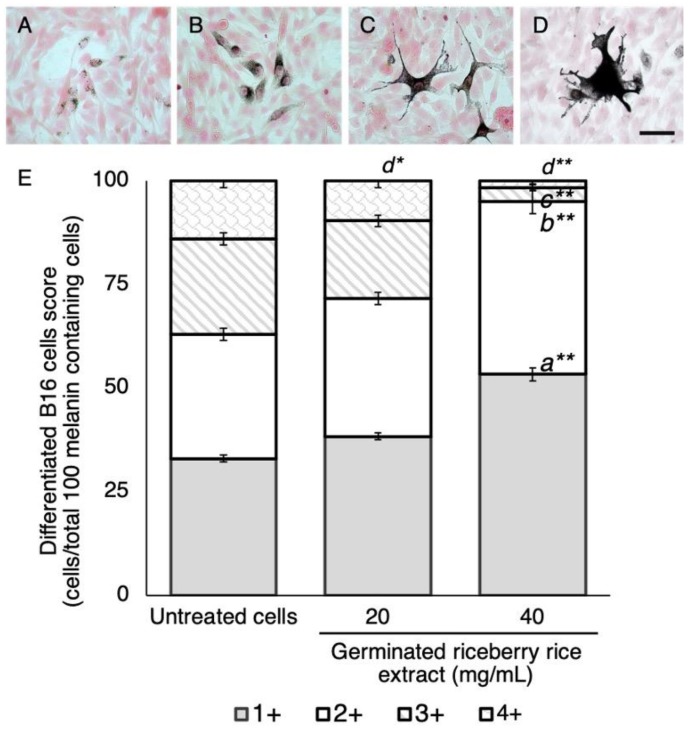
Effect of germinated riceberry rice extract on the morphological appearance of B16 cells. Differentiated B16 cells were scored as 1+ (**A**), 2+ (**B**), 3+ (**C**), and 4+ (**D**). Scoring was conducted by observing a total of 100 melanin containing cells under a light microscope at 100× magnification. Results were expressed as the mean ± SD from triplicate samples from two blinded independent experiments (**E**). *,** Statistical significances at *p* < 0.05 and *p* < 0.001 and *^a, b, c, d^* versus a score of 1+, 2+, 3+, and 4+ of untreated cells, respectively. Scale bar = 50 μm.

The morphological appearance of differentiated B16 cells is associated with their cellular melanin accumulation. In this study, the morphology of differentiated B16 cells was scored as 1+, 2+, 3+, and 4+ (Figure 3A–D). Germinated riceberry rice extract reduced the population of high score differentiated B16 cells to have a lower score in a dose dependent manner (Figure 3E). At 20 mg/mL of treatment, the number of 4+ differentiated B16 cells was significantly suppressed to 10 ± 2.05 cells/total 100 melanin containing cells when compared with untreated cells (14 ± 1.63 cells/total 100 melanin containing cells) (*p* = 0.049). The effect was shown more clearly at 40 mg/mL of treatment. The number of 3+ and 4+ differentiated B16 cells was significantly suppressed to 3 ± 1.25 and 2 ± 0.47 cells/total 100 melanin containing cells, respectively, when compared with untreated cells (23 ± 1.41 and 14 ± 1.63 cells/total 100 melanin containing cells, respectively) (*p* < 0.001). The results demonstrated that germinated riceberry rice extract not only reduced melanin biosynthesis, but also affected the morphological appearance associated with melanocyte differentiation. 

### 3.5. Melanin Excretion and Cellular Oxidants of B16 Cells Treated with Germinated Riceberry Rice Extract

**Figure 4:**
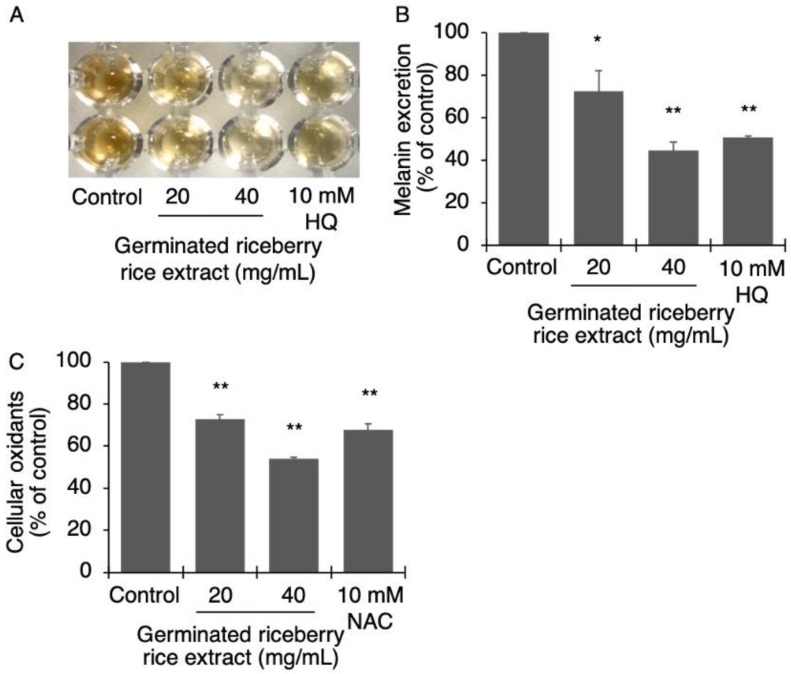
Effect of germinated riceberry rice extract on melanin excretion and cellular oxidants. B16 cells were treated with germinated riceberry rice extract at 20 and 40 mg/mL. The melanin excretion was optically determined in a culture medium (**A**). Results were expressed in a bar graph (**B**) as the percentage of melanin excretion by comparing the mean ± SD with 100% of untreated cells. Cellular oxidants were also determined (**C**). Ten micromolar hydroquinone (HQ) and N-acetylcysteine (NAC) were used as positive results for tyrosinase inhibition and antioxidants, respectively. *,** Statistical significances at *p* < 0.01 and *p* < 0.001, respectively, when compared with untreated cells. Results from triplicate assays of two independent experiments.

Melanin excretion is a spontaneous characteristic of differentiated B16 cells. Likewise, the treatment of B16 cells with germinated riceberry rice extract at the concentrations of 20 and 40 mg/mL significantly inhibited the melanin excretion in a dose dependent manner to 72 ± 9.87% (*p* = 0.001) and 45 ± 4.15% (*p* < 0.001), respectively, when compared with untreated cells (100%) (Figure 4A,B). 

The high antioxidant property of germinated riceberry rice extract was a target of tyrosinase-related melanogenesis inhibition. In this study, the cellular oxidants in B16 cells were then elucidated (Figure 4C). The results showed that germinated riceberry rice extract at the concentrations of 20 and 40 mg/mL significantly reduced cellular oxidants in B16 cells to 73 ± 2.105% (*p* < 0.001) and 54 ± 0.963% (*p* < 0.001), respectively, when compared with untreated cells (100%). 

### 3.6. Phytophenolics Fingerprints of Germinated Riceberry Rice Extract 

**Figure 5:**
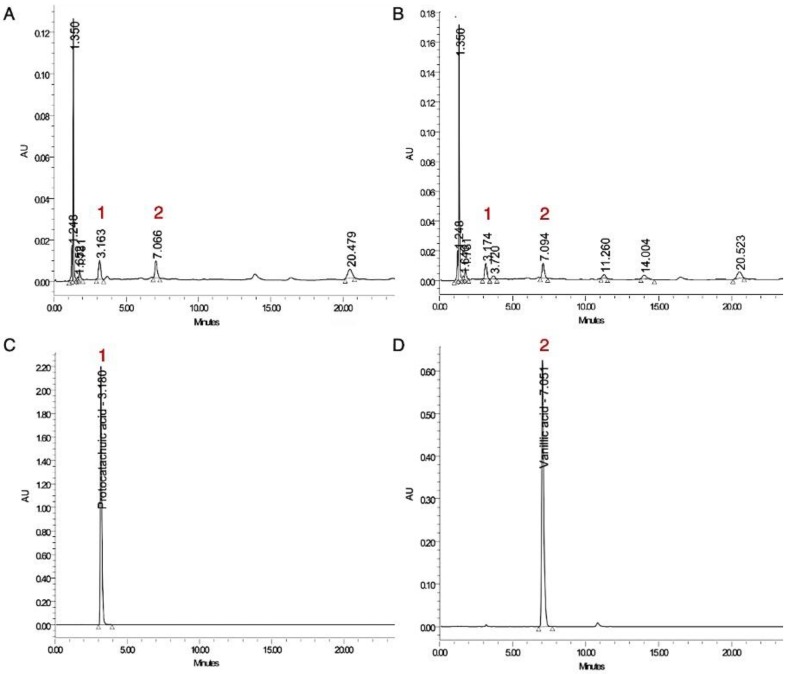
The HPLC chromatogram of ungerminated riceberry rice extract (**A**), germinated riceberry rice extract (**B**), standards **1**: protocatechuic acid (**C**) and **2**: vanillic acid (**D**).

Germinated riceberry rice extract had high antioxidants and also showed high potency for anti-melanogenesis. The inhibition was associated with tyrosinase activity and cellular oxidants. Thus, the predominant phytochemicals in germinated riceberry rice extract were then identified with a comparison to ungerminated riceberry rice extract (Figure 5A,B). The results showed that two increasingly predominant phenolic compounds were presented in germinated riceberry rice extract and displayed the percentage of Areas 1 and 2 at 3.87% and 2.20%, respectively, when compared with the total area. Meanwhile, the percentages of Areas 1 and 2 of ungerminated riceberry rice were 2.20% and 0.87%, respectively, when compared with the total area. Then, the two increasingly dominant peaks of 1 and 2 were identified compared with the standards as protocatechuic acid and vanillic acid, respectively. 

## 4. Discussion

The germination of riceberry rice promoted its antioxidants, while the phenolic contents were similar. As presented in a previous study, the short-term germination of the grain slightly altered the total phenolic content, but dynamically changed the phenolic profiles [26]. It is well known that tyrosinase enzyme plays a legitimate role in melanogenesis and can be modulated by antioxidants [8,27]. In this study, germinated riceberry rice extract had a strong inhibitory effect on tyrosinase-related melanogenesis and also affected the morphological appearance of B16 cells by reducing cell size, cellular melanin distribution, and dendriticity. In a previously study, the dendritic morphology was established as a hallmark of melanocyte differentiation-associated tyrosinase expression. It is also known to be the preceding process for melanin translocation [27,28]. In agreement with this study, germinated riceberry extract reduced the melanin excretion of B16 cells. In addition, cellular oxidative stress is recognized as a cause of hyperpigmentation and also used as a target for pigmentation control [29,30,31]. Concerning this study, germinated riceberry rice extract not only showed antioxidant activity in test tubes, but also reduced cellular oxidants in B16 cells. Furthermore, two dominant phenolic compounds were identified from germinated riceberry rice extract as protocatechuic acid and vanillic acid, which were previously established as an antioxidant and tyrosinase inhibitor [5,32]. These findings demonstrated that the short-term germination of riceberry rice promoted protocatechuic acid and vanillic acid, which may inhibit melanogenesis through the role of cellular oxidant-related tyrosinase activity. The extract also reduced cell differentiation-associated melanin excretion and showed potential for hyperpigmentation control.

## 5. Conclusions

These results demonstrated the potential of short-term germinated riceberry rice extract as a new source of antioxidants and tyrosinase inhibitors. The extract also effectively reduced the melanin production effectively by interfering with the differentiation of melanin producing cells. Two targeted phenolic compounds, protocatechuic acid and vanillic acid, which have been recognized as tyrosinase inhibitors, were dominantly promoted under this germinating condition.

## Figures and Tables

**Table 1:** Differentiated B16 cells scoring system.

Criterion	Score
Dendritic shape with a bipolar spindle, cell size at 1–50 μm, pigmentation lower than 50% of cytoplasmic area with a low distribution.	1+
2. Dendritic shape with a bipolar spindle, cell size at 1–50 μm, pigmentation equal to or more than 50% of cytoplasmic area with a higher distribution.	2+
3. Multi-polar spindle without tip branches, cell size larger than 51 μm. Pigmentation throughout the cytoplasmic area.	3+
4. Multi-polar spindle with numerous tip branches, cell size larger than 51 μm. Pigmentation densely packed throughout the cytoplasmic area.	4+

**Table 2:** Antioxidant capacity, total phenolic content, and mushroom tyrosinase activity.

Conditions	Antioxidant Capacity			Total Phenolic Content	Mushroom Tyrosinase Activity
	ABTS (mg Trolox Equivalent/g Sample)	DPPH (mg Vit C Equivalent/g Sample)	FRAP (M FeSO_4_·7H_2_O Equivalent/g Sample)	Folin–Denis (mg Gallic Acid Equivalent/g Sample)	Mushroom Tyrosinase Inhibition IC_50_ (mg/mL)
Ungerminated	1.37 ± 0.02	0.09 ± 0.01	3.05 ± 0.01	1.28 ± 0.01	123.26 ± 3.21
Germinated	2.08 ± 0.04 **	0.14 ± 0.01 *	3.29 ± 0.05 *	1.27 ± 0.01	60.08 ± 4.24 **

*,** Significant results when compared with ungerminated at *p* ≤ 0.05 and *p* < 0.001, respectively.
